# Hydroxyapatite-based bone repair biomaterials based on clinical heterogeneity: modification strategies, performance regulation, and personalized repair pathways

**DOI:** 10.3389/fbioe.2026.1896532

**Published:** 2026-08-24

**Authors:** Leiyun Huang, Jinghan Hu, Qingjin Cai, Jing Qian, Yuncheng Bai, Shangwei Wu, Yu Zhang, Yifan Wang, Hongrui Gao, Daiwei Wang, Zengdong Meng, Yongzhen Liu

**Affiliations:** 1 People’s Hospital of Wenshan Prefecture, Wenshan, China; 2 The Affiliated Wenshan Hospital of Kunming University of Science and Technology, Wenshan, China; 3 Medical School, Kunming University of Science and Technology, Kunming, China; 4 Wenshan Hospital of Traditional Chinese Medicine, Wenshan, China; 5 The Affiliated Wenshan Hospital of Yunnan University of Chinese Medicine, Wenshan, China; 6 Department of Urology, Urologic Surgery Center, Xinqiao Hospital, Third Military Medical University (Army Medical University), Chongqing, China; 7 The First People’s Hospital of Yunnan Province, Kunming, China

**Keywords:** biomaterial modification, bone defect repair, clinical heterogeneity, hydroxyapatite, personalized design

## Abstract

The repair of bone defects represents a major clinical challenge in orthopedics, oral and maxillofacial surgery, and trauma surgery. The heterogeneity of their etiology, anatomical sites, local microenvironments, and patients’ systemic conditions imposes diverse and sometimes contradictory performance requirements on repair materials. As the primary inorganic component of human bone, hydroxyapatite (HA) is considered an ideal foundational material for bone repair due to its excellent biocompatibility, osteoconductivity, and osteoinductive potential. However, its inherent brittleness, insufficient mechanical strength, and single functionality limit its application in complex clinical scenarios. This article reviews the latest advances in the modification of HA-based materials through strategies such as ion doping, composite reinforcement, structural regulation, and surface functionalization, aiming to precisely modulate their mechanical properties, degradation behavior, osteogenic activity, antibacterial capacity, and pro-vascularization functions. Furthermore, this article provides an in-depth analysis of the heterogeneous characteristics of various clinical bone defect types—including infectious, load-bearing, ischemic (insufficient blood supply), osteoporotic, and post-tumor resection defects—and their differential requirements for material performance. On this basis, a new clinical problem-oriented material design paradigm is proposed, shifting from a “universal” approach to a “personalized” one. This involves a personalized research and development strategy that conducts precise functional integration and performance trade-offs for specific clinical scenarios (e.g., “antibacterial-dominant,” “mechanical-bioactive synergy,” “osteogenesis/anti-resorption dual regulation,” “pro-vascularization induction,” and “integrated tumor suppression and repair”), thereby achieving a precise match between material properties and specific clinical needs. Finally, this article discusses the current limitations regarding the standardization of performance evaluation, multi-functional synergistic mechanisms, and clinical translation. It also anticipates future development directions for personalized bone repair materials, including the construction of a multi-scale and multi-dimensional material performance evaluation system, the advancement of “personalized responsive” material development, and the strengthening of clinical translation research.

## Graphical Abstract

**Figure:**
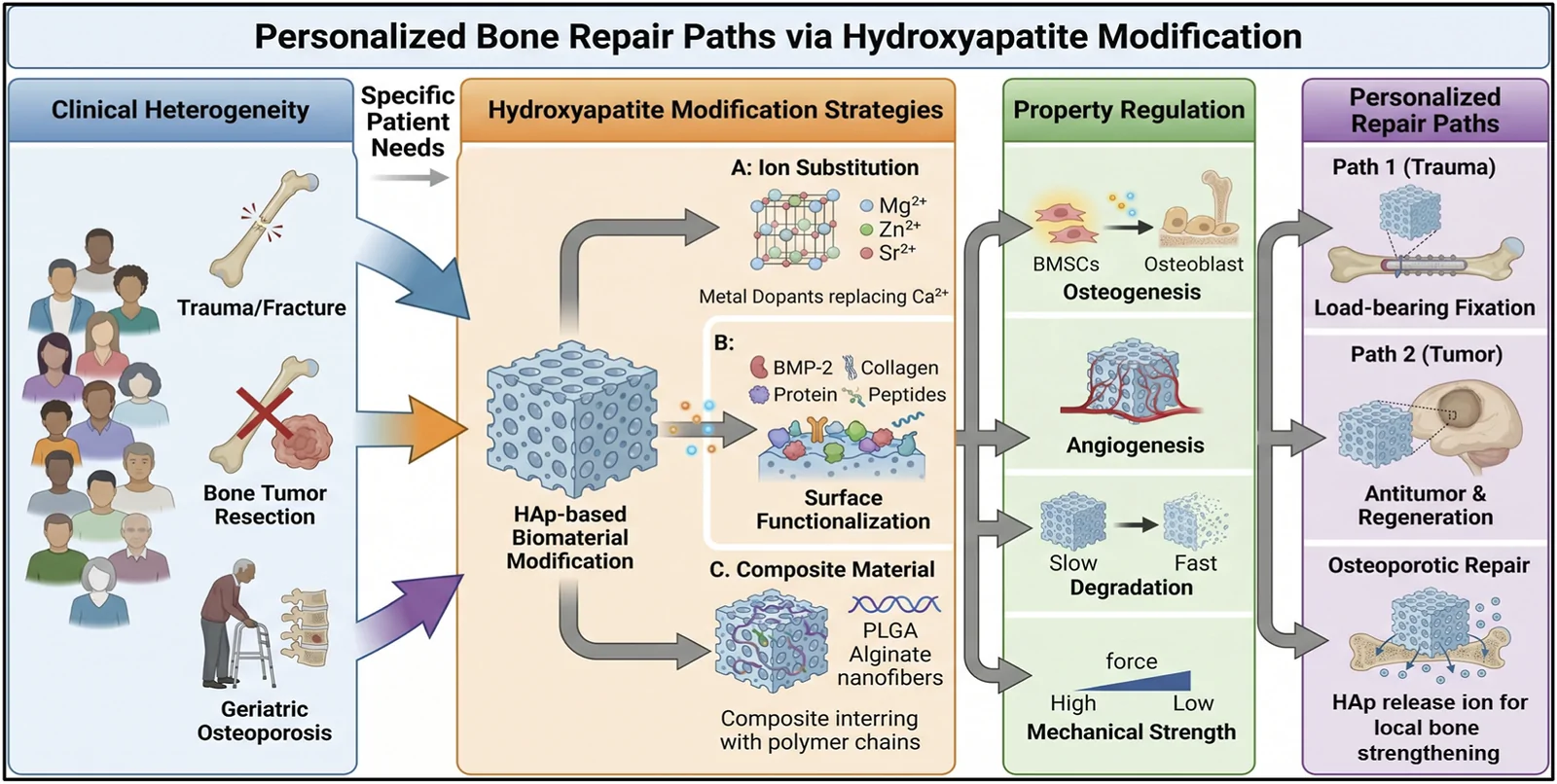

## 1 Introduction

The repair of bone defects presents a major clinical challenge in orthopedics, oral and maxillofacial surgery, and trauma surgery. Its incidence and the corresponding demand for treatment continue to escalate in tandem with an aging population and an increase in trauma events (Dinatha et al., 2024). Globally, the number of bone defect cases resulting from trauma, tumor resection, infection, and congenital anomalies is rising annually. Among these, critical-sized bone defects incapable of spontaneous healing typically require surgical intervention (Zhou et al., 2024). Autologous bone grafting, the current gold standard in clinical treatment, possesses inherent limitations including donor site morbidity and restricted availability of bone volume (Hu et al., 2020). Conversely, allogeneic bone grafting carries the risks of immune rejection and disease transmission (Luo et al., 2018; Ge et al., 2018). Furthermore, the majority of “universal” synthetic bone substitute materials available on the market frequently struggle to meet the diverse requirements of complex clinical scenarios, leading to suboptimal repair outcomes (Bian et al., 2026; Lai et al., 2021).

The root cause of this clinical dilemma lies in the profound heterogeneity of the bone defects themselves (Zhou et al., 2024; Deng et al., 2017). Ranging from trauma and infection to tumor resection, and from the highly vascularized jaw to the heavily load-bearing femur, variances in etiology, anatomical site, defect size, and host conditions (e.g., the presence of comorbidities such as osteoporosis) impose multifaceted and occasionally contradictory performance requirements on repair materials (Li et al., 2020). For instance, infectious defects urgently necessitate potent antibacterial capabilities (Yuan et al., 2020); defects in load-bearing regions require materials that provide matched mechanical support and an appropriate degradation rate (Hu et al., 2020); and osteoporotic defects require a dual-regulatory capacity to simultaneously promote osteogenesis and inhibit bone resorption (Kuo et al., 2025). These differentiated demands for material properties underscore the urgency and necessity of developing novel bone repair materials capable of precisely matching specific pathophysiological environments.

In this context, hydroxyapatite (HA), as the primary inorganic component of human bone, has consistently been regarded as an ideal foundational material for bone repair due to its innate biocompatibility, osteoconductivity, and inherent osteoinductive potential (Luo et al., 2018; Bian et al., 2026). However, the “intrinsic nature” of pure HA renders it inadequate for complex clinical applications: its inherent brittleness and inferior mechanical properties struggle to withstand the stresses in load-bearing regions (Luo et al., 2018); its degradation behavior may mismatch the growth rate of newly formed bone (Hu et al., 2020) and its functional “singularity” prevents it from independently addressing multifaceted challenges such as infection and insufficient blood supply (Deng et al., 2017; Yuan et al., 2020). Fortunately, advancements in materials science have provided diverse strategies for remodeling the properties of HA. Through ion doping (e.g., with strontium (Hu et al., 2020; Ge et al., 2018), magnesium (Deng et al., 2017), zinc (Fan et al., 2024), silicon (Lu et al., 2024), and silver (Yuan et al., 2020)), the crystallinity and degradation rate of HA can be significantly modulated, simultaneously endowing it with specific biological activities such as promoting osteogenesis, angiogenesis, or antibacterial properties. Composite and structural engineering strategies—such as integrating with polymers (e.g., PLLA (Ge et al., 2018), PCL (Maleki-Ghaleh et al., 2026)) to improve toughness, or constructing multi-scale porous structures to facilitate cellular ingrowth and mass exchange (Rath et al., 2025; Rath et al., 2024)—have further broadened the dimensions of its performance regulation. Furthermore, frontier research has combined HA with growth factors (e.g., rhBMP-2 (Deng et al., 2017)) or anti-tumor components (e.g., MoS_2_ (Huang et al., 2023a)) to construct multifunctional integrated systems responsive to specific microenvironments.

These advances reveal a clear paradigm shift: the design of bone repair materials is transitioning from the pursuit of a “one-size-fits-all” universal approach toward a clinical problem-oriented personalized era (Zhou et al., 2024; Huang et al., 2023a). This implies that future research and development must undertake precise functional integration and performance trade-offs tailored to specific clinical scenarios, such as “antibacterial-dominant infectious defects,” “mechanical-bioactive synergistic load-bearing defects,” “osteogenesis/anti-resorption dual-regulatory osteoporotic defects,” “pro-vascularization-inducing ischemic defects,” and “integrated tumor-suppressing and repairing post-tumor resection defects” (Kuo et al., 2025; Huang et al., 2023a; Huang et al., 2023b). Therefore, this article aims to review the latest advances in modifying HA-based materials through strategies including ion doping, composite reinforcement, structural regulation, and surface functionalization. It provides an in-depth analysis of the heterogeneous characteristics across different types of clinical bone defects and their differential requirements for material properties. Ultimately, it delineates a personalized repair pathway extending from “material properties” to “clinical efficacy,” with the goal of providing novel insights and strategies for the development of next-generation highly efficient, personalized, and precise bone repair solutions.

## 2 Clinical heterogeneity of bone defects and the demand for personalized mHA biomaterials

As the primary inorganic component of bone tissue, hydroxyapatite (HA) is considered an ideal foundational material for bone repair due to its excellent biocompatibility, osteoconductivity, and osteoinductive potential. However, its inherent brittleness, insufficient mechanical strength, and functional singularity limit its application in complex clinical scenarios. Clinical bone defects exhibit a high degree of heterogeneity, encompassing multiple variables such as infection, load-bearing requirements, blood supply status, and the patient’s systemic condition (e.g., osteoporosis). This imposes differentiated and occasionally contradictory performance requirements on repair materials. Therefore, the design of HA-based materials must transition from a “universal” approach to a “personalized” one, utilizing modified hydroxyapatite (mHA) to precisely address the repair challenges associated with diverse pathological characteristics (as shown in Table 1).

**TABLE 1:** Clinical Heterogeneity and Performance Requirements of mHA Materials.

Clinical bone defect type	Antibacterial capacity	Mechanical strength	Pro-osteogenesis	Pro-angiogenesis	Osteogenesis/Osteoclastogenesis dual regulation	Antitumor activity	Representative studies
Infectious bone defects	+	+	+	+	−	−	PCL/nano-HA aerogel (containing antibacterial components) (Deng et al., 2024); silver/nickel co-doped HA (Sivakumar et al., 2023); gallium-loaded HA/calcium carbsonate composite scaffolds (Piccinini et al., 2026)
Load-bearing bone defects	−	+	+	±	−	−	PA66/HA composites (Zeng et al., 2025); polyamino acid/nHA/high-strength and high-modulus PVA fiber composites (Wu et al., 2026)
Ischemic bone defects	−	±	+	+	−	−	Sodium alginate/nano-HA (Alg/n-HA) hydrogels (El-Kady et al., 2023); pro-vascularization and osteogenesis-coupled 3D printed scaffolds (Yin et al., 2026)
Osteoporotic bone defects	−	±	+	+	+	−	Micro/nano-hierarchical structured nwHA scaffolds (nHA-modified wHA) (Zhao et al., 2026); POL-modified HA scaffolds (activating the PI3K/AKT pathway) (Zhou et al., 2025)
Post-tumor resection bone defects	+	±	+	+	+	+	Bifunctional thermosensitive hydrogels (antibacterial and pro-osteogenic) (Tian et al., 2024); antitumor/immunomodulatory HA composites (Ma et al., 2022)

This table maps the differential performance requirements for modified hydroxyapatite (mHA) materials across five typical clinical bone defect types, illustrating the design paradigm shift from a “universal” to a “personalized” approach. By utilizing qualitative symbols (“+” indicates a core requirement, “−” indicates a non-primary requirement, and “±” indicates a selective requirement), the table explicitly delineates the prioritization of key properties—such as antibacterial capacity, mechanical strength, pro-osteogenesis, pro-vascularization, dual regulation, and antitumor activity—tailored to distinct pathological scenarios. Corresponding representative studies (e.g., PCL/nano-HA and PA66/HA composites) are also provided.

As the primary inorganic component of natural bone tissue (accounting for approximately 65%–70%), hydroxyapatite (HA) possesses the chemical formula Ca_10_(PO_4_)_6_(OH)_2_ and a Ca/P molar ratio of approximately 1.67. Its high similarity to human bone mineral endows it with excellent biocompatibility, osteoconductivity, and even a certain degree of osteoinductive potential (Moradi et al., 2021; Lim et al., 2020). Extensive animal studies have confirmed that synthetic hydroxyapatite effectively promotes new bone formation, mineralized matrix deposition, and cellular infiltration across various bone defect models, including rabbits, dogs, and rats (Xu et al., 2019; Rahimnia et al., 2021; Jang et al., 2017). For instance, in a rabbit femoral condyle defect model, simvastatin-loaded nano-hydroxyapatite scaffolds significantly increased bone mineral density and bone volume (Chalisserry et al., 2019); in a canine mandibular defect model, the application of Wishbone hydroxyapatite combined with a collagen membrane achieved a bone regeneration rate of 46.8% at 4 weeks, which was significantly superior to that of the commercial bovine bone mineral group (De Carvalho et al., 2024).

Before delving into specific material requirements, it is essential to establish the precise definition and boundaries of ‘clinical heterogeneity’ within the context of this study. Herein, ‘clinical heterogeneity’ refers to the diverse and highly variable physiological, pathological, and anatomical conditions presented by bone defects across different patients. The boundaries of this heterogeneity encompass not only the primary etiology (e.g., trauma, severe infection, or tumor resection) and the specific anatomical site (e.g., load-bearing regions such as the femur versus non-load-bearing regions such as the maxilla), but also extend to the local cellular microenvironment (such as vascularization status and inflammatory levels). Furthermore, systemic conditions and patient-specific variables—including patient age, sex, genetic background (e.g., congenital bone anomalies), and prior treatment history (e.g., radiotherapy, chemotherapy, or previous medical interventions)—must all be taken into consideration. While acknowledging the vastness of these intersecting variables, to provide highly targeted and translational guidance for material design, this review synthesizes this broad heterogeneity into five highly representative clinical pathological archetypes: infectious, load-bearing, ischemic, osteoporotic, and post-tumor resection defects. By delineating this non-exhaustive yet explicit boundary and subsequently categorizing it into distinct clinical scenarios, we establish that clinical bone defects cannot be regarded as a singular entity, thereby strictly defining the scope and focus of our personalized material design framework.

In cases of infectious bone defects, materials must simultaneously possess potent antibacterial properties and the capacity to promote bone regeneration. Studies have demonstrated that minocycline-loaded hydroxyapatite membranes can inhibit infection-induced bone resorption while simultaneously promoting mandibular regeneration (Wang et al., 2023). Furthermore, silver- or silicon-co-substituted hydroxyapatite coatings not only retard the proliferation of *Staphylococcus aureus* but also significantly upregulate the expression of osteogenic markers (Lim et al., 2017). However, the incorporation of antibacterial agents is frequently accompanied by the risk of cytotoxicity. For instance, although gold nanorod-functionalized magnesium-doped hydroxyapatite (MgHA) scaffolds achieved 100% bacterial eradication, they also resulted in a 4.38-fold reduction in cell numbers after 24 days (Franco et al., 2021), underscoring the critical challenge of balancing antimicrobial efficacy with biocompatibility.

For large segmental bone defects in load-bearing regions (e.g., the femur and tibia), materials must possess sufficient macroscopic mechanical strength for support. Under such circumstances, pure hydroxyapatite is inadequate; its compressive modulus must be enhanced by integrating reinforcing phases (e.g., graphene oxide or polymers). For instance, graphene oxide (GO)-enhanced strontium-substituted hydroxyapatite/chitosan scaffolds exhibited a compressive modulus of 438.5 kPa—four times that of pure polymer scaffolds—and significantly promoted bone regeneration in a rat calvarial defect model (Wu et al., 2020). Additionally, finite element analysis has revealed that the mechanical properties of magnesium-doped hydroxyapatite closely approximate those of human cortical bone (Bhatnagar et al., 2024).

In bone defect regions with insufficient blood supply (e.g., osteoradionecrosis and diabetic foot), promoting vascularization becomes critical. Human tooth-derived hydroxyapatite not only promotes bone regeneration in mouse calvarial defects but also demonstrates excellent vascularization-inducing capabilities (Lim et al., 2020). Furthermore, chitosan-loaded strontium-silk fibroin co-assembled hydroxyapatite microspheres have also exhibited the dual functions of ectopic osteogenesis and pro-angiogenesis in a subcutaneous model (Liu Y. et al., 2024).

The repair of bone defects in osteoporotic patients requires a balance between promoting osteogenesis and inhibiting osteoclastogenesis. Icariin-loaded hydroxyapatite/sodium alginate scaffolds can simultaneously upregulate osteogenic gene expression and inhibit osteoclast activity (Xie et al., 2019); meanwhile, magnesium-incorporated biomimetic collagen/nano-hydroxyapatite scaffolds, at an equivalent concentration of 25 mM, both enhance bone matrix deposition and reduce osteoclast maturation, demonstrating the dual-regulatory effect of ion release (Paiva et al., 2025).

Furthermore, the irregular large defects resulting from bone tumor resection demand materials that not only fill the anatomical space but also possess antitumor potential. The integrated design of “tumor suppression and repair” is emerging as a novel direction. Although current data may not directly provide extensive antitumor evidence, the application of hydroxyapatite as a drug carrier (e.g., miR-26a-loaded PLGA/HA scaffolds) has demonstrated the feasibility of synergizing controlled release with osteogenesis (Li X. et al., 2025), laying a solid foundation for future multifunctional integration.

The heterogeneity of clinical bone defects dictates that hydroxyapatite-based materials cannot rely on a “one-size-fits-all” design paradigm. Instead, they must be precisely engineered to modulate mechanical properties, degradation behavior, biological activity, and immunomodulatory functions in accordance with specific pathological characteristics, thereby achieving a paradigm shift from “universal” to “personalized” bone repair strategies (Sobczak-Kupiec et al., 2021; Dadvand et al., 2026; Shang et al., 2022).

## 3 Modification methods of hydroxyapatite-based biomaterials and their regulation of material properties

**FIGURE 1:**
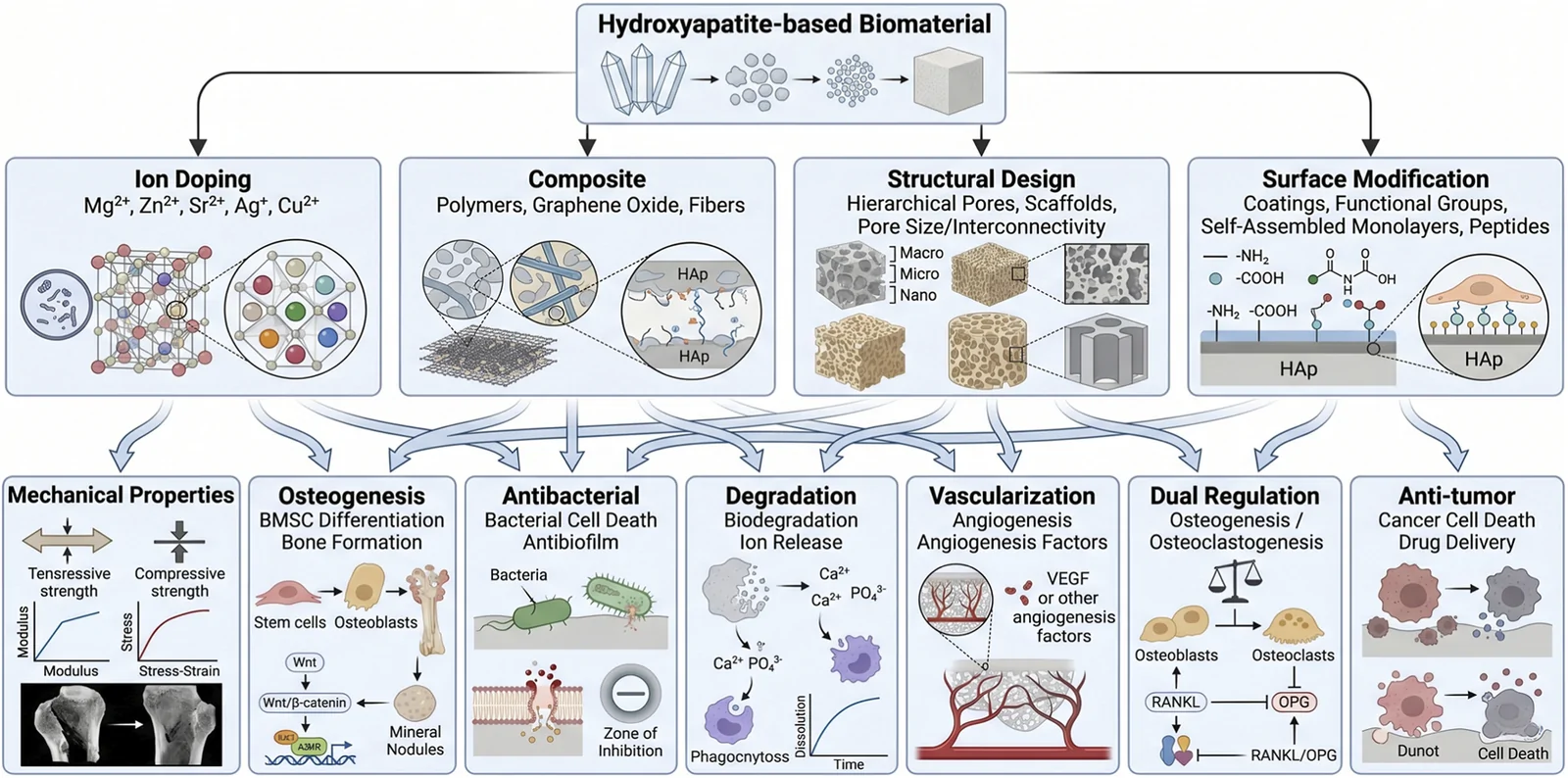
Mechanisms of HA-based biomaterial modification and property regulation for bone repair. As illustrated in figure, there are four key approaches to modify HA scaffolds: ion doping, creating composite materials, designing hierarchical structures, and altering surfaces. These engineering methods, through intricate and interconnected processes, enable precise control over seven essential characteristics for bone tissue engineering. These characteristics encompass mechanical strength, osteogenic capabilities, antibacterial effects, adjustable biodegradation, promotion of vascularization, dual modulation of osteogenic and osteoclastogenic functions, and targeted delivery of antitumor medications, among others.

To overcome the inherent limitations of HA and endow it with multifunctionality, researchers have developed various modification strategies. These primarily include: 1) ion doping (e.g., Sr, Mg, Zn, F) to modulate biological activity, antibacterial properties, or mechanical performance; 2) composite reinforcement (compounding with polymers, carbon materials, etc.) to enhance mechanical strength and toughness; 3) structural regulation (constructing hierarchical pores and micro/nano surfaces) to optimize cellular migration and nutrient diffusion; and 4) surface functionalization (loading bioactive molecules or drugs) to introduce specific functions such as antibacterial and pro-vascularization capabilities. These methods enable the precise regulation of the materials’ mechanical properties, degradation behavior, biological activity, and immunomodulatory functions, thereby establishing the materials science foundation for addressing complex clinical demands (as shown in Figure 1).

### 3.1 Modification methods and their regulation of material properties

To overcome the inherent limitations of HA and endow it with multifunctionality, researchers have developed various modification strategies. These primarily include ion doping, composite reinforcement, structural regulation, and surface functionalization. These methods enable the precise regulation of the materials’ mechanical properties, degradation behavior, biological activity, and immunomodulatory functions, thereby establishing the materials science foundation for addressing complex clinical demands.

Ion doping is a crucial means to enhance HA performance and biological activity. Fluorine (F^−^) doping significantly improves crystal structure stability and mechanical durability; specifically, 2% fluorine doping optimally enhances hardness and anti-degradation ability, though exceeding this amount (e.g., 3%) can conversely accelerate degradation due to increased porosity (Erdem et al., 2022). Magnesium (Mg^2+^) and zinc (Zn^2+^) co-doping regulates osteoblast behavior: Mg doping significantly promotes osteoblast spreading, proliferation, and osteocalcin production, while Zn doping enhances collagen I synthesis and extracellular matrix mineralization (Kazimierczak et al., 2019). Furthermore, strontium (Sr^2+^)-substituted HA reinforced with graphene oxide (GO) significantly increases the scaffold’s compressive modulus (reaching 438.5 kPa) and *in vitro* mineralization capacity (Wu et al., 2020). Doping with other elements, such as lanthanides (De Lama-Odria et al., 2023), chromium (Predoi et al., 2025), iron (Habib et al., 2024), and europium (Eu) (Liu et al., 2021), has also been utilized to modulate morphology, antibacterial properties—such as Fe, Zn, and Cr doping yielding a 14–16 mm inhibition zone against Gram-negative bacteria (Habib et al., 2024)—or fluorescent tracking functions while promoting osteogenic gene expression (Liu et al., 2021). It is important to note that while a myriad of trace elements have been explored for HA doping, this review deliberately limits its primary focus to ions (e.g., Sr^2+^, Mg^2+^, Zn^2+^, Ag^+^, Si^4+^) that have accumulated substantial high-level preclinical evidence.

Composite reinforcement strategies compensate for the mechanical shortcomings of HA by introducing second-phase materials. For example, compounding 3 wt% carbon nanotubes (CNTs) into HA can increase fracture toughness by over 200% while maintaining good biocompatibility (Siddiqui et al., 2018). Constructing hierarchical porous scaffolds with reduced graphene oxide (rGO) simultaneously provides robust mechanical strength and a degradation behavior that matches new bone formation (Zhou et al., 2019). Polymer matrices such as poly(lactic-co-glycolic acid) (PLGA), chitosan (CS), and quaternized chitosan (QCS) are also widely utilized to prepare HA composite scaffolds, which not only improves the flexibility of the materials but also enhances cell adhesion and biological activity (Wu et al., 2020; Gutierrez-Prieto et al., 2019; Pereira et al., 2020).

In terms of structural regulation and biomimetic synthesis, optimization significantly enhances cellular migration and load-bearing capacity. Strontium-doped HA ceramics featuring a micro/nano-composite surface significantly elevate the alkaline phosphatase (ALP) activity of bone marrow mesenchymal stem cells and promote *in vivo* bone and blood vessel regeneration (Jiang S. et al., 2022). The ultralong hydroxyapatite nanowires (UHANWs) strategy effectively overcomes the inherent high brittleness of traditional HA, achieving high deformability and elasticity (Zhu and Lu, 2019). Additionally, biomimetic synthesis pathways—utilizing natural raw materials like marine organism-derived calcium carbonate and bovine bone—preserve natural microstructures and enhance mechanical properties; for instance, the biaxial flexural strength of bovine bone-derived HA ceramics can reach 109.0 MPa with an elastic modulus of 105.17 ± 14.65 GPa (Gutierrez-Prieto et al., 2019; Piras et al., 2024; Minim et al., 2023).

Surface functionalization endows HA with additional capabilities, such as antibacterial and immunomodulatory functions. Loading tannic acid (TA) into HA-zirconia composites significantly enhances antibacterial and antioxidant capabilities (Yeasmin et al., 2025). Although nano-functionalization with gold nanorods (AuNRs) increases antibacterial efficiency to 100%, vigilance regarding long-term cytotoxicity is still required (Franco et al., 2021). Modifying HA with aminopropyltrimethoxysilane and crosslinking with chitosan achieves efficient drug adsorption and rapid release (Pereira et al., 2020). Furthermore, bioactive molecules such as platelet-rich fibrin (PRF) (Ye et al., 2023) and fibrin sealants (Pagani et al., 2025) can be integrated via coprecipitation or coating to regulate the immune microenvironment, such as guiding macrophage polarization to construct a favorable osteoimmune microenvironment (Shang et al., 2022).

Advanced Biological and Multifunctional Regulation: Beyond fundamental mechanical and structural enhancements, targeted modifications precisely orchestrate advanced biological responses. For instance, biomimetically synthesized amino acid-modified HA can induce the osteogenic differentiation of human mesenchymal stem cells without the need for exogenous growth factors (Payne et al., 2016). HA/Ag nanocomposites effectively inhibit multiple pathogens (such as *Escherichia coli* and *S. aureus*) at non-cytotoxic concentrations while simultaneously reducing the secretion of inflammatory factors (Martinez-Sanmiguel et al., 2019). Furthermore, the amorphous calcium phosphate content in HA composites can be regulated by Platelet-Poor Plasma (PPP) co-precipitation, thereby affecting their solubility and degradation profile (Glazov et al., 2023). Advanced immunomodulatory strategies, such as the combined use of vitamin D3 and MK-7, can also alleviate HA-induced oxidative stress, maintaining cellular redox balance and mitigating lipid peroxidation (Ambrozewicz et al., 2019).

Ultimately, these modification methods have transitioned from single-property optimization toward multifunctional integration, precisely regulating material properties to lay the materials science foundation for addressing complex clinical demands (Rial et al., 2021; Fendi et al., 2024; Bhat et al., 2022).

### 3.2 Molecular biological mechanisms induced by mHA material modification

**FIGURE 2:**
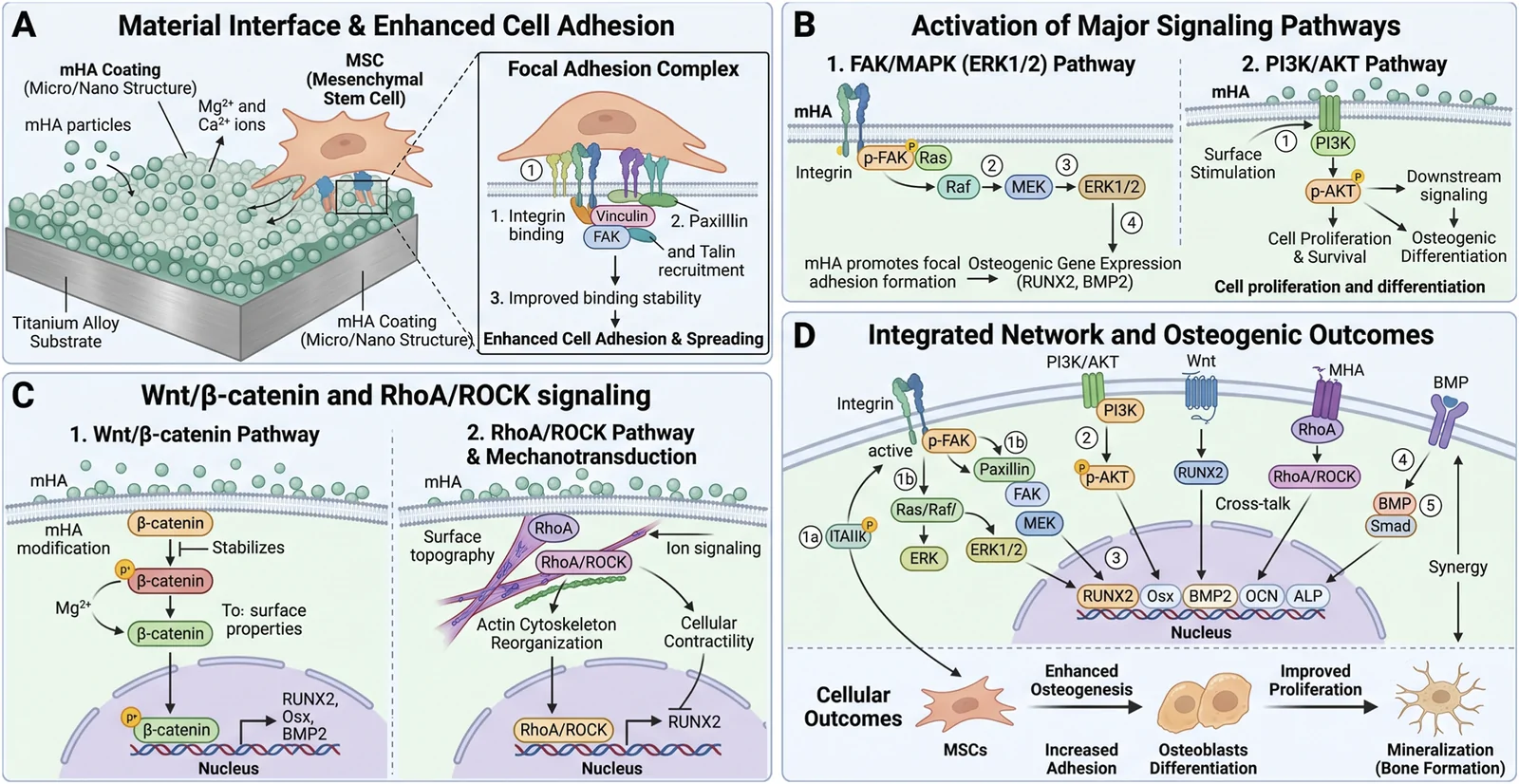
Molecular mechanisms of osteogenesis induced by mHA material modification. In Figure, Molecular biological mechanisms and signaling networks underlying osteogenesis induced by modified hydroxyapatite (mHA) materials. This schematic illustrates how mHA materials trigger multi-layered osteogenic biological responses through precise regulation of cell–material interfacial events. First, the micro-/nanotopography of the mHA surface and the release of bioactive ions (e.g., Mg^2+^, Ca^2+^) improve the local microenvironment, enhance early recognition by mesenchymal stem cells (MSCs), and promote the formation of stable focal adhesion complexes **(A)**. Subsequently, these physicochemical stimuli are transduced inward via transmembrane receptors, activating not only the FAK/MAPK and PI3K/AKT pathways that regulate cell proliferation and early osteogenic gene expression **(B)**, but also the Wnt/β-catenin pathway, which serves as a central hub for osteogenesis, as well as RhoA/ROCK mechanotransduction signaling mediating cytoskeletal reorganization **(C)**. Ultimately, multiple signaling networks — including Integrin, PI3K/AKT, Wnt, RhoA/ROCK, and BMP/Smad — undergo intensive cross-talk and synergy within the cell, potently upregulating core transcription factors such as RUNX2 and Osx as well as late-stage osteogenic markers (OCN, ALP), collectively driving the complete osteogenic cascade from stem cell differentiation to bone matrix deposition and mineralization **(D)**.

Physicochemical modifications of hydroxyapatite (HA)-based materials precisely regulate cell–material interfacial molecular events, triggering multi-level biological responses (as shown in Figure 2).

Transcriptomic analyses have confirmed that nano-HA (nHA) coatings significantly upregulate the expression of key factors such as BMP-2 and VEGF in osteoblasts, providing molecular evidence for material-induced osteogenesis and angiogenesis (Ogawa et al., 2026).

In terms of osteogenic mechanisms, HA directly enhances osteogenic differentiation by activating the mTOR signaling pathway (Bu et al., 2025). Strontium/zinc co-doped mesoporous silica nanoparticles (SrZn-MSNs) synergistically activate the PI3K-AKT pathway via integrins (Itgβ8, Itgα4) and Toll-like receptor 2 (Tlr2), markedly promoting osteogenic differentiation of bone marrow mesenchymal stem cells (BMSCs) (Cui et al., 2025). The synergistic action of VEGF and calcium ions regulates the directed differentiation of BMSCs through the VEGFR2/PI3K/AKT axis, offering a novel target for vascular–osteogenic coupling (Liu X. et al., 2024).

In angiogenic regulation, the hypoxic microenvironment activates the HIF-1α signaling pathway in macrophages, driving early vascular network formation—a mechanism experimentally confirmed in tricalcium phosphate (TCP)-induced bone regeneration (Bai et al., 2022). QK peptide-functionalized magnesium coatings mimic VEGF ligands, significantly enhancing CD31 expression, endothelial cell migration, and tubular structure formation. When nano-HA is incorporated into GelMA hydrogels loaded with ginsenoside R1 (NGR1), it synergistically upregulates VEGF/VEGFR-2 expression, activates the AMPK/HIF-1α pathway, and alleviates oxidative stress, thereby substantially promoting rat calvarial defect repair (Tan et al., 2026). The Wnt/β-catenin pathway serves as a central hub for osteogenesis–angiogenesis coupling; its activation simultaneously promotes bone formation and vascular invasion while inhibiting osteoclast activity.

Immunomodulation represents a critical dimension in functional material design. Biomimetic HA coatings suppress the release of pro-inflammatory cytokines such as TNF-α while promoting the secretion of BMP-2 and VEGF, thereby optimizing the macrophage polarization microenvironment (Jiang J. et al., 2022). Hydroxyapatite nanoparticles (HANPs) modulate macrophage phenotype switching by enhancing TLR4 signal transduction. Meanwhile, natural biomaterial-based tissue-engineered bone exhibits “temporal immunomodulatory” characteristics—initially eliciting moderate pro-inflammatory responses and later shifting toward an anti-inflammatory phenotype to accelerate bone regeneration. Mechanistically, material surface properties can inhibit p38-MAPK pathway activity, reduce NF-κB and JAK-STAT inflammatory signaling intensity, and guide macrophage polarization toward the M2 phenotype via metabolic reprogramming (Chen et al., 2024).

Regarding antibacterial functionality, nano-silver-modified HA exerts antimicrobial effects through the FoxO signaling pathway; however, the accompanying ROS accumulation and PPARγ pathway activation may mildly interfere with osseointegration (Bu et al., 2025). Novel peptide-functionalized HA scaffolds achieve surface-specific binding of antimicrobial peptides, inhibiting *S. aureus* and *E. coli* while maintaining VEGF expression and angiogenic capacity. Notably, material-induced immune microenvironment dysregulation (e.g., aberrant NLRP3 inflammasome activation) may impede vascularization and bone regeneration (Galaz et al., 2023), underscoring the need for a delicate balance between antibacterial activity and immune homeostasis.

### 3.3 *In vivo* metabolism and long-term biosafety of mHA

The *in vivo* metabolic behavior and long-term biosafety of modified hydroxyapatite (mHA)-based bone repair materials are pivotal prerequisites for successful clinical translation. Existing studies have confirmed that mHA exhibits excellent biocompatibility and osteoconductivity due to its high compositional similarity to natural bone mineral (Durairaj et al., 2026; Shaikh et al., 2024); however, critical challenges persist regarding its *in vivo* degradation kinetics and reciprocal interactions with host metabolism. The physical properties of the material significantly influence host metabolic responses: micro- and nano-sized HA particles can differentially drive macrophage metabolic reprogramming, wherein micro-sized particles induce alterations in mitochondrial dynamics and a glycolytic shift to promote a pro-inflammatory (M1) phenotype, whereas nano-sized particles do not trigger equivalent effects, suggesting a size-dependent immunometabolic regulatory mechanism (Shanley et al., 2023).

Regarding *in vivo* degradation, conventional HA exhibits slow resorption rates attributable to its high crystallinity and low solubility (Lu et al., 2023), whereas material modifications can optimize metabolic matching. For instance, Mg(Ti-HA) nanocomposites demonstrate a degradation rate of up to 32% within 60 days in a Wistar rat model, synchronously elevating the bone regeneration rate from 10% to 15% at 30 days to 60%–70% at 60 days; similarly, low-crystalline carbonated HA (L-CHA) displays superior biodegradability compared to conventional HA, as its structural profile more closely resembles physiological biological apatite (Lu et al., 2023).

However, significant knowledge gaps remain in long-term biosafety evaluations. Although studies explicitly indicate that ion-doped HA (e.g., doped with Zn, Sr) can enhance antibacterial performance and modulate macrophage polarization toward an anti-inflammatory M2 phenotype to optimize the pro-regenerative microenvironment (Wu et al., 2023), the release kinetics, *in vivo* biodistribution, and potential cytotoxicity of these doping ions have not yet been evaluated (Furlani et al., 2024). Furthermore, the genotoxic risks and long-term immunomodulatory impacts of nano-HA remain unresolved issues. While hydroxyapatite offers distinct advantages as an antibiotic carrier for localized drug delivery, its subchronic systemic toxicity and systemic metabolic impacts following long-term implantation “have not been fully explored.”

Most current *in vivo* investigations are largely confined to short-term observation windows (e.g., 60 days) and lack robust follow-up data extending beyond 18 months. Moreover, there is a severe scarcity of mechanistic research investigating the elimination pathways of degradation products, the risks of multi-organ bioaccumulation, and their correlations with inter-individual metabolic heterogeneity (such as baseline renal function status). Moving forward, it is imperative to integrate multi-scale metabolic tracking technologies to establish a long-term biosafety evaluation framework tailored to clinical requirements, thereby overcoming the critical bottlenecks hindering the bench-to-bedside clinical translation of mHA materials.

## 4 Internal trade-offs and multi-dimensional balancing in the performance regulation of mHA biomaterials

**FIGURE 3:**
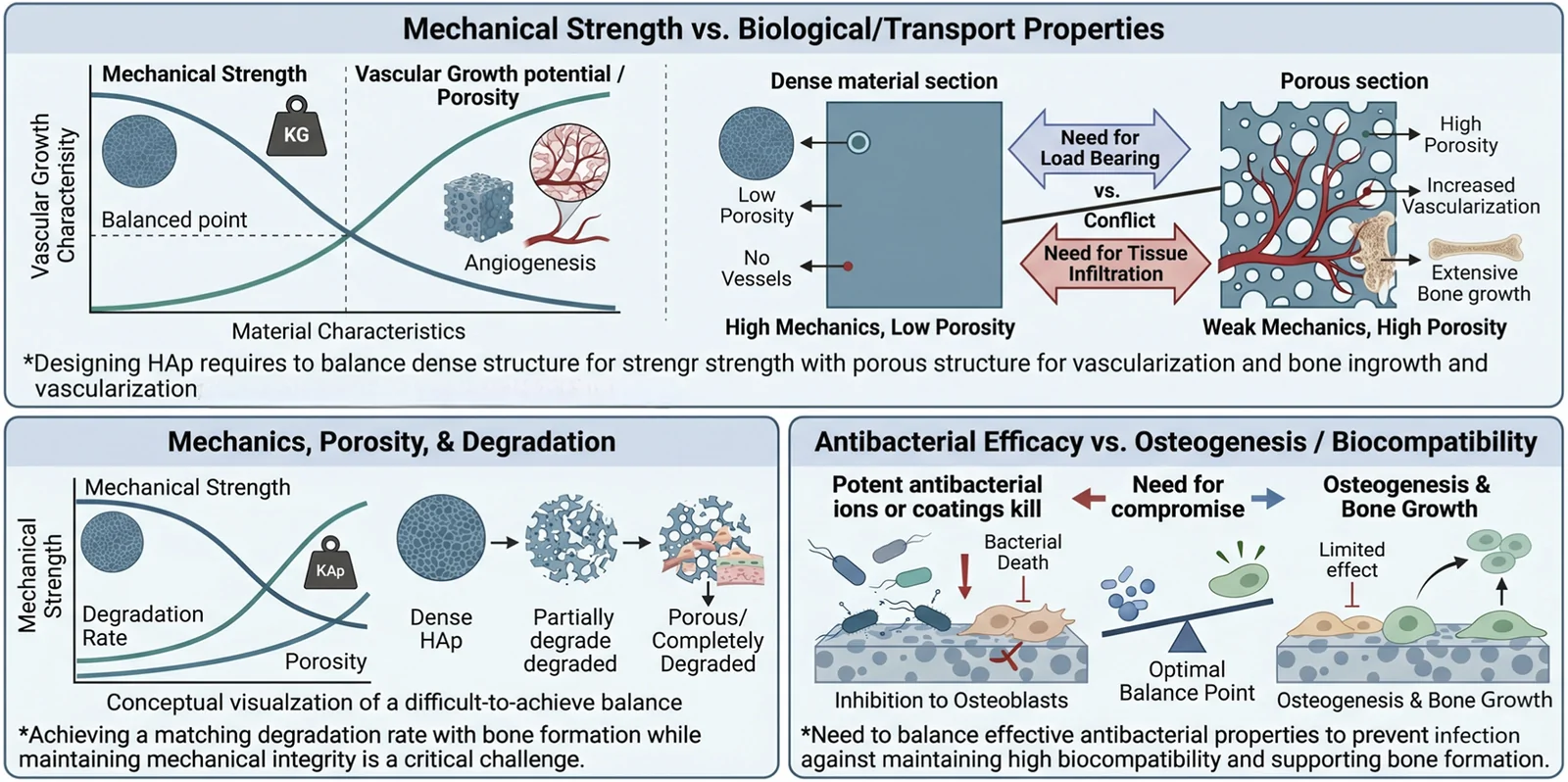
Conceptual view of performance regulation trade-offs in HA-based biomaterials. Figure illustrates crucial internal equilibria necessary for the optimization of biomaterials. The first balance highlights the tension between mechanical strength and biological or transport characteristics. While dense structures enhance load-bearing capacity, they hinder vascularization and tissue integration, which are facilitated by increased porosity. The second equilibrium pertains to the relationship among mechanical stability, porosity, and the rate of degradation, indicating the challenge of aligning material degradation with the formation of new bone. The third balance addresses the tradeoff between antibacterial properties and osteogenic or biocompatibility effects, suggesting that the application of potent antibacterial agents or coatings must be carefully calibrated to avoid harming osteoblasts.

In the pursuit of optimizing material properties, a mutually restrictive “seesaw effect” frequently emerges among different performance indicators. The core contradictions include the following: 1. Macroscopic mechanical strength versus microscopic porous/vascularization space: Enhancing mechanical strength frequently results in decreased porosity, thereby compromising cellular infiltration and nutrient exchange. 2. Antibacterial capacity versus osteogenesis/biocompatibility: The incorporation of potent antibacterial agents may introduce cytotoxicity, necessitating a delicate balance between bacterial inhibition and the promotion of tissue regeneration. 3. Degradation rate versus the pace of bone regeneration: A material that degrades too rapidly fails to provide sustained mechanical support, whereas excessively slow degradation impedes the substitution by new bone. 4. Multifunctional integration: When integrating multiple properties—such as mechanical support, antibacterial activity, and pro-vascularization—the various functional modules may interfere with one another. Consequently, personalized design necessitates seeking a systemic, optimal trade-off across these multi-dimensional properties (as illustrated in Figure 3).

### 4.1 The mutual constraint between macroscopic mechanical support strength and microscopic porous networks and vascular infiltration space

The core advantage of hydroxyapatite (HA) as a bone repair material lies in its excellent biocompatibility and osteoconductivity, but its inherent brittleness and low fracture toughness severely restrict its application in load-bearing regions. To enhance macroscopic mechanical properties, researchers frequently improve compressive strength, flexural strength, and fracture toughness by introducing reinforcing phases (such as carbon nanotubes, graphene, metal oxides, or polymers). For example, incorporating 3 wt% carbon nanotubes can increase the fracture toughness of HA composites by over 200% (Siddiqui et al., 2018); meanwhile, after vacuum sintering, the flexural strength of graphene-doped HA ceramics can reach the level of natural cortical bone (Li Q. et al., 2025). However, such reinforcement strategies are often accompanied by a decrease in porosity and densification of the microstructure, thereby restricting cellular migration, nutrient diffusion, and vascular ingrowth. Studies have shown that although the addition of 4.0% silicate nanoclay significantly increases the Young’s modulus to 199 MPa, it simultaneously reduces porosity and degradation rate (Yadav and Verma, 2023); similarly, although 3D-printed scaffolds with high HA content (>80%) possess inorganic composition and mechanical properties approaching those of human bone, the construction of their porous structure relies on whiskers or polymer additives to maintain a certain degree of pore interconnectivity (Zhao et al., 2024). Furthermore, deproteinized HA obtained through low-temperature sintering degrades rapidly due to its high porosity and large specific surface area, but its low density and poor crystallinity result in insufficient mechanical support capacity (Ramirez Fernandez et al., 2017). Therefore, while pursuing high strength, it is essential to simultaneously consider pore structure design to ensure adequate space for vascular infiltration and tissue regeneration, revealing a distinct trade-off relationship between the two.

### 4.2 The balance between antibacterial properties and osteogenesis/biocompatibility

Infection is a major cause of failure in bone defect repair, making the endowment of HA materials with antibacterial functions an urgent clinical demand. Common strategies include doping with silver, zinc, or fluorine, or introducing photothermally active components (such as graphene). For example, under 808 nm near-infrared irradiation, the surface temperature of graphene-modified HA can exceed 60 °C, achieving a bacterial inhibition rate of 96% while promoting the proliferation and differentiation of bone marrow mesenchymal stem cells; its dual efficacy of anti-infection and osseointegration has also been validated in a rabbit femoral infection model (Li Q. et al., 2025). Silver-doped HA coatings also exhibit significant antibacterial activity; the 3% Ag-HAP coating not only improves hardness and wear resistance but also maintains good biocompatibility (Kumar et al., 2025). However, the introduction of antibacterial agents may negatively impact cellular behavior. For instance, after loading silver nanoparticles, although HA/MWCNT composites exhibit a strong inhibitory effect on various bacteria and fungi (with inhibition zone diameters reaching 18–20 mm), their specific surface area decreases from 109.4 m^2^/g to 35.3 m^2^/g, which may affect protein adsorption and cell adhesion (Unal, 2025). In addition, although fluorine doping can enhance the crystal stability and mechanical properties of HA, when the doping amount exceeds 2%, it conversely leads to increased surface porosity, multiplied structural defects, and accelerated degradation, thereby weakening its long-term biological stability (Erdem et al., 2022). Thus, the reinforcement of antibacterial functions requires strict control over dosage and release kinetics to avoid sacrificing osteoinductivity and cytocompatibility, achieving a precise balance of “inhibiting bacteria without inhibiting growth.”

### 4.3 The spatiotemporal matching contradiction among mechanical strength, degradation rate, and porosity

Ideal bone repair materials should provide initial mechanical support while gradually degrading in tandem with new bone formation, achieving a dynamic match between mechanical properties and tissue regeneration. However, HA itself degrades slowly, making it difficult to meet this spatiotemporal synergistic requirement. Although modification strategies can regulate degradation behavior, they frequently conflict with mechanical properties. For example, nHA/mHA coatings significantly enhance the compressive strength retention of magnesium-based implants after 6 weeks of immersion in simulated body fluid (86%–90% vs. 66% in the uncoated group) and improve stem cell adhesion, demonstrating a good coordination of mechanics, degradation, and biocompatibility (Tian et al., 2019); conversely, deproteinized HA prepared at low sintering temperatures experiences excessively rapid *in vivo* degradation due to low crystallinity and a reduction in the calcium-to-phosphorus ratio to 1.08 ± 0.32, rendering it unable to provide sustained support (Ramirez Fernandez et al., 2017). On the other hand, although the addition of biodegradable polymers such as PLGA or PCL can adjust the overall degradation rate, it may weaken the initial strength. For instance, although HA/Si/PLGA composites maintain 96% osteoblast viability, their mechanical properties primarily rely on the PLGA matrix, limiting their long-term stability (Gutierrez-Prieto et al., 2019). Notably, hierarchical pore structure design can partially alleviate this contradiction: HA/rGO composite scaffolds, constructed with suitable pore size and porosity via a soft template method, match their degradation rate with new bone formation while maintaining robust biomechanical strength, significantly accelerating bone ingrowth (Zhou et al., 2019). Therefore, presetting the synchronization of the “strength attenuation curve” and the “bone regeneration curve” during the material design stage remains a critical challenge.

### 4.4 The “seesaw effect” of multifunctional integration

Modern bone repair materials tend to integrate multiple functions, including mechanical support, antibacterial properties, pro-vascularization, osteoinduction, and controllable degradation; however, mutual constraints frequently exist among these functional modules, forming a typical “seesaw effect.” For example, increasing the HA content or introducing rigid fillers (such as TiO_2_ orAl_2_O_3_) to enhance strength often reduces the flexibility and processability of the material; although a high HA content (>80%) is conducive to osteoconduction, the elevated slurry viscosity affects 3D printing precision (Rstakyan et al., 2024). As another example, carboxylic acid-functionalized polycarbonate block copolymers can extend the antibiotic release of bone cement against *S. aureus* to 259 days and increase the total porosity by threefold (from 4.3% to 12.5%), but it remains necessary to ensure that mechanical strength is not compromised (Liang et al., 2021). Furthermore, although graphene simultaneously contributes to mechanical reinforcement, photothermal antibacterial activity, and the promotion of cell differentiation, it may trigger side reactions during high-temperature sintering, generating new phases such as Ca_3_(PO_4_)_2_ and TiP, which conversely reduce densification and increase brittleness (Li et al., 2018). In addition, although multifunctional ion co-doping (e.g., Mg, Zn, Sr, Li) can synergistically improve mechanical properties and biological activity (Fendi et al., 2024), the optimal concentration window for each ion differs, and excessive amounts may lead to cytotoxicity or structural instability (De Lama-Odria et al., 2023). These instances demonstrate that the extreme optimization of a single property often comes at the expense of other functions. Only through systemic material design, the construction of gradient structures, and intelligent response mechanisms (such as 4D printing) (George et al., 2022) can an optimal trade-off point be found among multi-dimensional properties, thereby achieving truly personalized bone repair.

### 4.5 Prioritization principles and the translational gap in design thresholds

A key bottleneck in current biomaterials research is that the precise quantitative upper and lower bounds of these performance trade-offs remain undefined in the literature. For instance, precise quantitative answers are still lacking to questions such as “What is the minimum acceptable compressive strength when maximizing porosity for load-bearing defects?” or “How much osteogenic activity can be safely sacrificed to achieve the requisite antibacterial efficacy?” The primary purpose of highlighting this “seesaw effect” in the present review is to foreground this major translational dilemma. The absence of standardized, scenario-specific design thresholds currently impedes the clinical translation of modified hydroxyapatite materials. This, therefore, represents an urgent call to action for the biomaterials community: future studies must shift from qualitative investigation toward quantitative inquiry—standardized *in vivo* studies aimed at defining precise quantitative boundaries and acceptable trade-off thresholds for specific clinical scenarios are critically needed.

## 5 Clinical heterogeneity and demand-driven personalized design strategies

**FIGURE 4:**
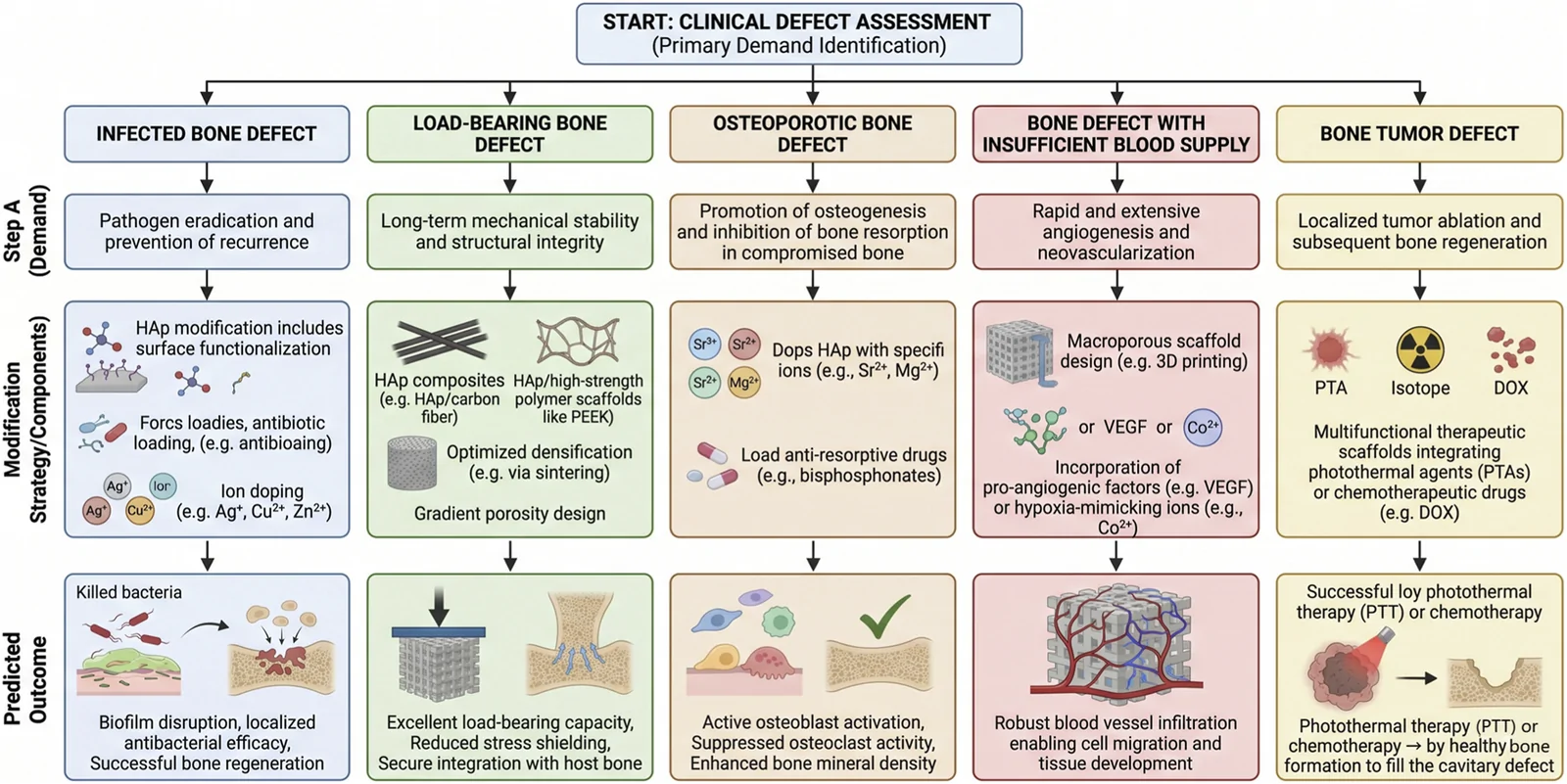
Personalized Repair Pathways for mHA Biomaterials. Figure illustrates various material engineering approaches aimed at assessing bone defects in a clinical context. It addresses five key clinical situations: infectious defects, load-bearing issues, osteoporotic conditions, defects with inadequate blood supply, and bone tumors, outlining their primary therapeutic needs. Following this, it summarizes the tailored modification strategies for HA. Among the strategies mentioned are surface functionalization, antibacterial and ion loading, high-strength composites designed with gradient porosity, specific ion doping, and the delivery of antiresorptive medications, as well as macroporous scaffolds incorporating pro-angiogenic factors and multifunctional platforms for photothermal and chemotherapeutic agents. Finally, it presents the anticipated clinical outcomes from these customized designs, which aim to achieve precise antibacterial effects, mechanical compatibility, regulation of the bone microenvironment, enhanced vascularization, and in situ tumor ablation in conjunction with bone regeneration.

To translate this concept into a practical operational framework, we have constructed a demand-driven decision workflow (Figure 4). This framework links the primary demands of five typical clinical scenarios with targeted HA modification strategies (e.g., specific ion doping, surface functionalization, or composite design) and their anticipated biological outcomes. Guided by this workflow, the following subsections analyze the pathological characteristics of each defect category and detail their corresponding personalized, modification-based repair pathways.

### 5.1 Infectious bone defects: antibacterial-led multifunctional material design

Infectious bone defects are typically secondary to osteomyelitis, open fractures, or postoperative infections. Their repair process requires not only the reconstruction of bone structure but also the effective control of the local pathogenic microbial load. Although traditional hydroxyapatite (HA) materials possess excellent biocompatibility and osteoconductivity, they are prone to becoming a breeding ground for bacterial colonization in infectious environments, which limits their application. Addressing such clinical scenarios, HA-based biomaterials must transition towards an antibacterial-led, multifunctional design that incorporates highly efficient antibacterial capabilities while accommodating osteoinductive and immunomodulatory functions. Existing studies have demonstrated that constructing HA composite scaffolds equipped with controlled drug release systems can simultaneously achieve bacterial inhibition and bone repair within the infectious microenvironment, supporting the single-stage treatment of infectious bone defects (Farazin and Mahjoubi, 2024). Furthermore, nano-hydroxyapatite/graphene oxide composite coatings on titanium alloy surfaces have exhibited excellent antibacterial activity and corrosion resistance while concurrently enhancing hydrophilicity and biocompatibility, providing a novel perspective for the prevention of implant-related infections (Vignesh et al., 2025). Recent studies have also shown that functional modification can endow HA materials with antibacterial capabilities without compromising their osteogenic performance. For example, after specifically binding the antimicrobial peptide PSI10 to the surface of 3D-printed porous HA scaffolds, the material exhibited potent antibacterial effects against both *S. aureus* and *E. coli* BL21. Simultaneously, it significantly promoted the expression of osteogenesis-related genes (*COL1A1*, *BGLAP*, *RUNX2*) and alkaline phosphatase activity (Zhongxing et al., 2021). Furthermore, under infectious conditions, HA-containing asymmetric membranes can still effectively promote the expression of osteogenesis-related genes (*RUNX2*, *SPP1*, *ALP*) and mineralization in MC3T3-E1 cells, and guide bone regeneration in a mandibular defect model (Wang et al., 2023). Cobalt-doped hydroxyapatite (COHA) has also been proven to possess antibacterial properties while simultaneously promoting osteoblast growth and reducing inflammatory responses (Lin et al., 2020). Lanthanide doping has also been confirmed to endow HA with potential dual functions of antibacterial activity and tissue regeneration by regulating its morphology and physical properties (De Lama-Odria et al., 2023).

### 5.2 Large segmental bone defects in load-bearing regions: synergistic design of mechanical properties and biological activity

Bone defects in load-bearing regions (e.g., the femur, tibia, and acetabulum) impose significantly higher demands on the mechanical properties of repair materials compared to non-load-bearing regions (e.g., the calvaria and mandible). Clinical data indicates that although a mixture of 65% HA and 35% β-tricalcium phosphate (TCP) can effectively repair bone defects caused by benign bone tumors, its integration speed is relatively slow and its mechanical strength is low, suggesting that it is more suitable for non-load-bearing or low-stress regions (Dragosloveanu et al., 2020). Therefore, for large segmental bone defects in load-bearing regions, a “mechanics-bioactivity synergistic” design is required to elevate mechanical properties while preserving or enhancing osteogenic capacity. In recent years, a variety of reinforcement strategies have been utilized. For example, 3D-printed poly(L-lactic acid)/hydroxyapatite (PLLA/HA) composite scaffolds significantly enhance interfacial compatibility through the modification of HA nanoparticles with dopamine and hexamethylenediamine, thereby achieving excellent mechanical properties and biocompatibility (Yang et al., 2018). In another study, polycaprolactone/borate bioglass (PCL/BBg) composite grafts demonstrated a compressive strength of approximately 9 MPa and an elastic modulus of 168 MPa in a rabbit anterior maxillary defect model, approaching the level of human mandibular cancellous bone, and promoted early osteogenesis without causing inflammatory or toxic responses (Saha et al., 2026). Furthermore, the compressive modulus of graphene oxide-enhanced strontium-doped hydroxyapatite/chitosan composite scaffolds reached 438.5 kPa, which is four times that of the control group, and significantly promoted bone regeneration in a rat calvarial defect model (Wu et al., 2020). Additionally, a 3D-printed biomimetic porous titanium alloy scaffold combined with a hydrogel microsphere system loaded with endothelial cell-derived exosomes not only improved bone volume and density but also significantly promoted neovascularization in a rabbit bone defect model (Luo et al., 2025). Silk fibroin/selenium-doped hydroxyapatite composites, owing to their porous, loose structure and excellent osteogenic capacity, have also been recommended for the repair of large segmental bone defects (Wang et al., 2017).

### 5.3 Bone defects with insufficient blood supply: pro-vascularization-induced material design

Insufficient blood supply is one of the key factors leading to delayed healing or failure of bone defects, commonly seen in conditions such as diabetes, osteoradionecrosis, or large segmental bone defects. Because breaking the vicious cycle of “ischemia-repair impairment” is critical, the core focus of material design for these defects lies in “pro-vascularization induction.” Multiple studies have shown that HA-based materials can improve local microcirculation by compounding with pro-angiogenic factors or elemental doping. For example, chitosan-strontium-silk fibroin-hydroxyapatite composite microspheres (CS/Sr-SF-HA) not only induced osteogenic differentiation in a rat subcutaneous implantation model but also exhibited significant ectopic osteogenic capacity, suggesting their pro-vascularization potential (Liu Y. et al., 2024). Furthermore, GGA-ZSP hydrogels significantly accelerated the bone repair process in a large segmental bone defect model by simultaneously promoting osteogenic differentiation and vascular regeneration (Wan et al., 2024). Hydroxyapatite-pyroxene composites also demonstrated the dual effects of promoting new bone formation and vascular regeneration in Sprague-Dawley (SD) rat calvarial defects; Microfil perfusion analysis confirmed that their degree of vascularization was superior to that of single-component materials (Ge et al., 2019). Flavonoid-loaded polymer implants significantly promote angiogenesis and osteogenesis by upregulating the expression of VEGF and CD31 and activating pathways such as Wnt, p38, AKT, and Erk (Yang et al., 2023). In addition, bone organoid units based on silk fibroin hydrogel microspheres effectively ameliorate the hypoxic microenvironment in mouse models by introducing oxygen-releasing components and immune cells, promoting M2 macrophage polarization and angiogenesis (Deng et al., 2025). 3D-printed scaffolds combined with hypoxia-induced exosomes also significantly increase neovascular density in rabbit models by activating the HIF-1 and VEGF pathways (Luo et al., 2025).

### 5.4 Osteoporotic bone defects: dual-regulation material design for osteogenesis and anti-resorption

Under osteoporotic conditions, the destruction of bone microarchitecture and the imbalance of bone turnover (enhanced osteoclast activity and weakened osteoblast activity) significantly increase the difficulty of bone defect repair. In such cases, merely providing an osteoconductive scaffold is insufficient to achieve effective regeneration. Consequently, a “dual regulation of osteogenesis and anti-resorption” strategy must be implemented to actively balance bone metabolism. One study seeded human OPG gene-modified bone marrow mesenchymal stem cells (BMSCs) onto HA scaffolds for use in an ovariectomy-induced osteoporotic rat mandibular defect model; the results showed that the composite significantly inhibited osteoclastogenesis and promoted new bone formation (Liu et al., 2016). Another study developed core-shell structured HA/BCP bioceramic particles that not only promoted BMSC proliferation and osteogenic differentiation but also inhibited the differentiation of RAW264.7 cells into osteoclasts, achieving a higher bone volume fraction and bone mineral density in critical-size femoral defects (Wu et al., 2022). Zn/Sr co-doped hydroxyapatite nanoparticles (ZnSr-HA) were also proven to synergistically promote early extracellular matrix formation and late osteogenic maturation, upregulating the expression of COL I and *BGLAP* (Durairaj et al., 2026). Strontium-doped hydroxyapatite/silk fibroin (SrHA/SF) composite nanospheres significantly enhance mesenchymal stem cell viability and osteogenic differentiation capacity *in vitro*, manifested by the upregulated expression of genes such as *RUNX2*, *ALP*, *COL1A1*, and *SPP1*, without compromising biocompatibility (Wang et al., 2020). Chitosan-strontium chondroitin sulfate (CH-SrCS) scaffolds effectively promote bone regeneration in aged rat models by downregulating the mRNA expression associated with inflammation and osteoclastogenesis while upregulating BMP2 levels (Xu et al., 2021). Furthermore, reviews indicate that HA-based materials targeting osteoporotic defects can achieve the controlled release of factors such as BMP-2 and VEGF through functional modification, and when combined with stem cells and extracellular vesicles, can construct scaffold systems with intelligent immune-responsive capabilities (Puthiyoth et al., 2026).

### 5.5 Bone defects following bone tumor resection: “integrated tumor suppression and repair” material design

Bone tumor resection often leaves large-volume, irregular bone defects accompanied by a disrupted local microenvironment (e.g., persistent inflammation, vascular destruction, and the potential risk of tumor recurrence). Clinical studies have shown that using a mixture of 65% HA and 35% TCP for repairing defects post-benign bone tumor resection achieved a 100% healing rate within 12 months with no observed complications, proving its efficacy as an autologous bone substitute (Dragosloveanu et al., 2020). However, for malignant or aggressive tumors, it is necessary to further integrate antitumor strategies. Therefore, ideal repair materials must employ an “integrated tumor suppression and repair” design, simultaneously accounting for structural reconstruction, biocompatibility, and the localized neutralization of malignant cells. Metal-organic frameworks (MOFs), due to their high specific surface area, controllable degradability, and drug-loading capacity, have been explored for constructing HA composite systems possessing both antitumor and bone regeneration functions (Zhao et al., 2023; Yun et al., 2025). Selenate-substituted hydroxyapatite (SeHA) nanoparticles exhibit the capability to inhibit osteosarcoma cell proliferation, reduce PTEN gene promoter methylation levels, and decrease tumor recurrence and metastasis both *in vivo* and *in vitro*, with the SeHA-0.01 dose group achieving a favorable balance between tumor-suppressive effects and nephrotoxicity (Wang et al., 2019). Silk fibroin/selenium-doped hydroxyapatite (SF/HASe) composites not only stably and slowly release selenate ions and increase cell proliferation activity but are also suitable for postoperative osteosarcoma treatment (Wang et al., 2017). Furthermore, Zn/Sr co-doped hydroxyapatite nanoparticles exhibit advantages in both early and late osteogenic stages by synergistically promoting cell attachment, proliferation, and matrix mineralization, suggesting their application potential in postoperative bone tumor defect repair (Durairaj et al., 2026). Finally, HA scaffolds combined with stem cell therapies (such as SHED or BMSCs) have also demonstrated the effects of enhancing bone regeneration and inhibiting osteoclastic activity (Saskianti et al., 2025; Tseng et al., 2024), suggesting they may play an active role in regulating the immune microenvironment following tumor resection.

## 6 Current controversies, limitations, and conflicting evidence in mHA research

Although current research on modified hydroxyapatite (mHA)–based bone repair materials has achieved notable progress in functional optimisation, multiple controversies and evidence gaps have emerged against the backdrop of clinical heterogeneity.

### 6.1 Mechanistic controversies and conflicting biological responses

Contradictions within ion-doping strategies are prominent. In silver-doped systems, high concentrations or unevenly distributed AgNPs enhance antibacterial performance yet provoke mammalian cytotoxicity; moreover, different synthetic routes (e.g., reverse-precipitation) produce markedly divergent silver distribution patterns and biological outcomes, and the trade-off threshold between antibacterial efficacy and biocompatibility remains unsettled (Kucukosman et al., 2025). Magnesium doping exhibits a concentration-dependent dual effect in 2D cultures—25 mM suppresses osteoclasts but concurrently inhibits osteoblast differentiation, while 50 mM induces cytotoxicity; intriguingly, the same concentrations in biomimetic scaffolds instead promote bone matrix deposition and suppress osteoclast maturation via synergy, suggesting that simplified *in vitro* models poorly predict *in vivo* responses within complex microenvironments, and the optimal release dosage and therapeutic window remain unclear (Paiva et al., 2025). Lithium-doped mHA upregulates alkaline phosphatase activity in C2C12 cells and hMSCs yet suppresses this marker in human osteoblasts (hOB), and the suppressive effect may originate from surface topography rather than ion release per se—highlighting the decisive role of cell-type heterogeneity in material response (Salam and Gibson, 2022).

Mechanistic disputes surround nanoparticle geometry. Spherical nHA (200 µM) induces S-phase arrest, apoptosis, and SULT1A3-mediated DNA-adduct pathway activation in HUVECs, whereas rod-shaped particles promote proliferation; although *in vivo* gavage assays show no marked organ toxicity, blood and metabolic parameters are already perturbed, casting doubt on the consistency between *in vitro* and *in vivo* findings and on the generalisability of geometry–effect relationships (Sun et al., 2024). Micron-scale HA drives macrophage glycolysis and provokes an M1 inflammatory phenotype, while nanoscale particles lack this effect; yet functionalised elongated rod-like HA effectively polarises macrophages toward M2 and promotes bone regeneration (Shanley et al., 2023; Luo et al., 2026)—the mechanistic link among geometry, surface modification, and immunometabolic regulation remains to be disentangled.

Tensions between degradation kinetics and regenerative efficacy are equally striking. Si-HA/BCP accelerates bone healing owing to rapid degradation (within 21 days), whereas the slowly degrading Si-HPMC/BCP leads to suboptimal repair—directly challenging the traditional dogma that “slower degradation favours mechanical support” (Flegeau et al., 2021). Low-crystalline carbonated HA (L-CHA) shows superior degradability and osteogenic activity, yet the precise role of carbonate within the lattice remains a black box (Lu et al., 2023), and a generalisable model for the dynamic matching between scaffold degradation rate and bioactive factor release (e.g., miR-29b) is still lacking (Pan et al., 2022).

### 6.2 Evidentiary discontinuities and the hierarchy of evidence

Biosafety assessment suffers from evidentiary discontinuities. A systematic review reports 20 positive outcomes among 71 genotoxicity assays, significantly correlated with needle-/rod-like nanostructures, yet evidence on point-mutagenicity is missing and long-term chronic exposure risks remain unresolved (de Souza et al., 2024). Zebrafish embryo models confirm no embryotoxicity or neurotoxicity for ZnHA, SrHA and other ion-doped microspheres, but such short-term models cannot capture systemic accumulation months or years post-implantation; metabolic risk assessment for special populations (e.g., renal insufficiency) is virtually blank.

Hierarchy of Evidence and Translational Gaps: A critical assessment of the current literature reveals a significant disparity in the strength and quality of evidence supporting mHA strategies. While highly advanced functionalizations (e.g., multi-ion co-doping, MOFs, and smart responsive hydrogels) demonstrate remarkable efficacy in in vitro cellular assays and *in vivo* small-animal models (predominantly murine and leporine), high-quality clinical evidence remains exceedingly scarce. The majority of clinically validated data is restricted to simple, first-generation modifications (e.g., basic HA/TCP biphasic mixtures) utilized in non-critical or benign bone tumor defects. Consequently, many of the ‘personalized’ strategies proposed herein currently rely on preclinical evidence (equivalent to Level IV or Level V evidence in the clinical hierarchy). Bridging this translational chasm requires future research to deliberately escalate the hierarchy of evidence. It is imperative to advance beyond small-animal screening and prioritize validation in large-animal models (e.g., ovine or porcine load-bearing defect models) under physiological biomechanical conditions, ultimately culminating in rigorously designed, multicenter randomized controlled trials (RCTs) across heterogeneous human populations.

Beyond the hierarchy of evidence, engineering and clinical-translation bottlenecks are particularly severe. Most studies are confined to single animal models (e.g., rabbit calvarial defects) or small cohorts (e.g., 16 rats) (Hefzollesan et al., 2026), lacking stratified validation across heterogeneous populations such as diabetic patients, elderly osteoporotic subjects, and developing individuals (e.g., children with cleft lip/palate). The absence of standardised preclinical models undermines data comparability, while engineering challenges—inter-batch consistency at scale, sterilisation compatibility, and synchrony between degradation and osteogenesis—remain unsolved (Li et al., 2026). Mechanistic contradictions such as mutual suppression in Sr–Mg co-doping, and the propensity of Zn/Sr co-doping to form unfavourable secondary phases, together with insufficient evidence on the stability of immunomodulatory functions (e.g., FPHA-induced macrophage polarization) across diverse pathological microenvironments (Chen et al., 2024), collectively point to a fragmented understanding of material–host–disease interplay in current research. Future work urgently calls for multi-centre, long-term, stratum-stratified validation frameworks that embed clinical heterogeneity into material design logic, so as to bridge the translation gap from “lab-effective” to “clinically deployable.”

### 6.3 Manufacturing scalability and regulatory readiness

Reproducibility, scalability, and regulatory readiness are critical gaps hindering the widespread clinical application of mHA materials (Moon et al., 2025; Ciccone et al., 2026). At the manufacturing level, minor fluctuations in synthesis processes can significantly affect material purity, crystallinity, and pore structure—for instance, scaffolds are prone to developing unintended pores during fabrication, further compromising their already limited load-bearing capacity. The stringent purity requirements combined with the limitations of current synthetic techniques collectively constrain batch-to-batch consistency in large-scale production, while rigorous control over quality, reliability, and traceability has yet to be established under unified industry standards (Safitri et al., 2026). Safety assessment also raises concerns: although nano-hydroxyapatite shows promise in dental remineralization, its biosafety and long-term biocompatibility remain questioned; short-term observations from preclinical models (e.g., rabbit tibial defects) are insufficient to predict long-term responses within the complex human microenvironment, and metabolic risk assessments for special populations such as those with renal insufficiency are virtually absent (Owji et al., 2024).

The dynamic evolution of regulatory pathways presents another challenge. Instances of medical device regulatory reclassification—such as China’s reclassification of red laser devices as Class III high-risk products following reports of clinical retinal damage—serve as a warning: when material functions are expanded (e.g., loading with antibiotics, growth factors, or imparting smart-responsive properties), the regulatory classification may escalate from ordinary implantable devices to drug-device combination products or high-risk biologics (Ostrin and Schill, 2026). Such reclassification demands more rigorous independent safety verification, long-term toxicological data, and full-chain quality control evidence across the production process. Yet most current mHA studies remain at the stage of basic performance characterization, lacking a standardized validation system that meets regulatory requirements (Rossello-Gelabert et al., 2026).

Regulatory readiness must encompass six dimensions: core capabilities, organizational culture, value proposition, technological foundation, policy compliance, and operational resources (Ciccone et al., 2026). However, existing evidence indicates that academic institutions exhibit significantly lower readiness in clinical and regulatory domains compared to theoretical research environments (de Jong et al., 2024); evidence for routine clinical implementation is scarce, and gaps persist in training, governance, interoperability, and regulatory oversight. Moreover, if mHA materials are defined as “manufactured products” (e.g., endowed with new functions through surface modification or composite strategies) rather than traditional biological extracts, the quality control logic must be restructured to satisfy drug regulatory authorities’ stringent requirements for reproducibility and batch stability (Rossello-Gelabert et al., 2026).

Although the U.S. FDA’s approval of poly(sebacic acid citrate)/hydroxyapatite degradable bone fixation devices marks a milestone, this case precisely underscores that regulatory clearance is achieved only when materials design, chemistry, manufacturing and controls (CMC), quality-by-design (QbD) strategies, and multi-center validation data form a closed loop. Looking ahead, integrating regulatory science upfront into the material design phase and employing a dual-track assessment of Technology Readiness Level (TRL) and Clinical Readiness Level (CRL) will help bridge the gap between laboratory innovation and clinical translation, thereby enabling safe, effective, and accessible personalized applications of mHA materials across heterogeneous clinical scenarios.

## 7 Conclusion and prospects

Bone defects exhibit significant heterogeneity in etiology, anatomical location, and local microenvironment, which necessitates that repair materials meet diverse requirements in terms of biological functionality, mechanical compatibility, and degradation kinetics. In recent years, hydroxyapatite (HA)-based biomaterials have made significant progress in the field of bone tissue engineering. Grounded in the core challenge of clinical heterogeneity, this review summarizes the performance requirements and modification strategies of hydroxyapatite-based materials across different bone defect scenarios, bridging the theoretical and practical gap in the transition from “universal materials” to “personalized repair.” By integrating the pathological characteristics of five typical defect categories—infectious, load-bearing regions, insufficient blood supply, osteoporotic, and post-tumor resection—targeted material design paradigms are proposed: such as antibacterial-led multifunctional integration, mechanics-bioactivity synergy, dual regulation of osteogenesis and anti-resorption, pro-vascularization induction, and integrated “tumor suppression and repair” strategies. This lays the foundation for establishing a personalized repair system geared toward complex, real-world bone defects.

However, although current research on hydroxyapatite-based materials has yielded fruitful results, existing evaluations mostly rely on single indicators (e.g., cell survival rate, ALP activity, or new bone formation rate), lacking a comprehensive evaluation framework for the multi-dimensional performance of mechanics, biology, and immunomodulation. Furthermore, differentiated testing standards applicable to various clinical scenarios (e.g., infectious, load-bearing, or osteoporotic defects) have not yet been established, which severely restricts their clinical translation and horizontal comparison. Secondly, significant differences exist among various studies regarding doping concentration, composite ratio, pore structure, degradation rate, and the loading methods of bioactive molecules, making it difficult to reproduce or integrate the results. For example, although zinc-doped HA promotes osteogenesis at low concentrations, its optimal release mechanism and safety threshold have not yet been clarified (Dornelas et al., 2024); although magnesium doping can improve compressive stress and fracture toughness, a concentration of 25 mM inhibits osteoblast differentiation (Paiva et al., 2025), indicating a narrow dosage window and a lack of standardized definition. In addition, long-term *in vivo* safety data are scarce; in particular, the metabolic pathways, accumulation effects, and impacts on the immune microenvironment of doped ions or nanocarriers remain unclear. For instance, although the introduction of antibacterial modifications such as silver, zinc oxide, or gold nanorods enhances bactericidal efficiency, it is accompanied by potential cytotoxicity risks—such as AuNRs-functionalized scaffolds resulting in a 4.38-fold decrease in cell number at 24 days (Franco et al., 2021).

Therefore, the future development of hydroxyapatite-based bone repair biomaterials should focus on three major directions: first, constructing a multi-scale, multi-dimensional performance evaluation system encompassing mechanical stability, degradation kinetics, immunomodulatory capacity, vascular induction efficiency, and long-term biosafety, alongside formulating differentiated standards tailored to different clinical subtypes; second, deepening the research and development of “personalized responsive” materials, utilizing technologies such as 3D printing and tunable degradation to enable materials to dynamically adapt to microenvironmental changes at various stages of bone regeneration; third, strengthening clinical translational research, particularly the need to conduct large-sample, multicenter human trials to validate the efficacy and safety of personalized design strategies in the real world. The ultimate goal is to achieve an “on-demand customized” bone repair solution—that is, precisely matching hydroxyapatite-based materials with specific mechanical, biological, and immunomodulatory characteristics according to the patient’s bone defect type, systemic condition, and local microenvironment, truly advancing toward a new paradigm of personalized regenerative medicine.

## Glossary

AgNPs: Silver Nanoparticles
AKT: Protein Kinase B
Al₂O₃: Aluminum Oxide
ALP: Alkaline Phosphatase (protein/activity)
ALPL: Alkaline Phosphatase (gene)
AMPK: AMP-activated Protein Kinase
AuNRs: Gold Nanorods
BBg: Borate Bioglass
BCP: Biphasic Calcium Phosphate
BGLAP: Bone Gamma-Carboxyglutamate Protein (Osteocalcin gene)
BMP-2 / rhBMP-2: (Recombinant human) Bone Morphogenetic Protein-2
CD31: Cluster of Differentiation 31
CH-SrCS: Chitosan-Strontium Chondroitin Sulfate
CNTs / MWCNT: Carbon Nanotubes / Multi-Walled Carbon Nanotubes
COHA: Cobalt-doped Hydroxyapatite
COL1A1: Collagen Type I Alpha 1 Chain (gene)
CS: Chitosan
Erk: Extracellular Signal-Regulated Kinase
FoxO: Forkhead Box O
FPHA: Fluorinated Hydroxyapatite
GelMA: Gelatin Methacryloyl
GGA-ZSP: Gallic Acid-Gelatin composite hydrogel with Zinc-Strontium Phosphate
GO / rGO: Graphene Oxide / Reduced Graphene Oxide
HA: Hydroxyapatite
HANPs / nHA: Hydroxyapatite Nanoparticles / Nano-Hydroxyapatite
HIF-1α: Hypoxia-Inducible Factor 1-alpha
JAK-STAT: Janus Kinase-Signal Transducer and Activator of Transcription
L-CHA: Low-Crystalline Carbonated Hydroxyapatite
MAPK / p38: Mitogen-Activated Protein Kinase
MgHA: Magnesium-doped Hydroxyapatite
mHA: Modified Hydroxyapatite
miR-26a / miR-29b: MicroRNA-26a / MicroRNA-29b
MOFs: Metal-Organic Frameworks
MoS₂: Molybdenum Disulfide
mTOR: Mammalian Target of Rapamycin
NF-κB: Nuclear Factor kappa B
NGR1: Notoginsenoside R1
NLRP3: NLR Family Pyrin Domain Containing 3
nwHA: Micro/Nano-hierarchical structured Hydroxyapatite
OPG: Osteoprotegerin
PA66: Polyamide 66
PCL: Polycaprolactone
PI3K: Phosphoinositide 3-Kinase
PLGA: Poly(Lactic-co-Glycolic Acid)
PLLA: Poly(L-Lactic Acid)
POL: Polydatin
PPARγ: Peroxisome Proliferator-Activated Receptor Gamma
PPP: Platelet-Poor Plasma
PRF: Platelet-Rich Fibrin
PTEN: Phosphatase and Tensin Homolog
PVA: Polyvinyl Alcohol
QCS: Quaternized Chitosan
RUNX2: RUNX Family Transcription Factor 2 (gene)
SeHA / HASe: Selenate-substituted / Selenium-doped Hydroxyapatite
SF: Silk Fibroin
Si-HPMC: Silanized Hydroxypropyl Methylcellulose
SPP1: Secreted Phosphoprotein 1 (Osteopontin gene)
SrHA: Strontium-substituted / doped Hydroxyapatite
Sr-mnHAp: Strontium-doped Micro/Nano-composite Hydroxyapatite
SrZn-MSNs: Strontium/Zinc Co-doped Mesoporous Silica Nanoparticles
SULT1A3: Sulfotransferase Family 1A Member 3
TA: Tannic Acid
TCP: Tricalcium Phosphate
TiO₂: Titanium Dioxide
TLR2 / TLR4: Toll-Like Receptor 2 / Toll-Like Receptor 4
TNF-α: Tumor Necrosis Factor-alpha
UHANWs: Ultralong Hydroxyapatite Nanowires
VEGF: Vascular Endothelial Growth Factor
VEGFR2: Vascular Endothelial Growth Factor Receptor 2
